# The correlation between maternal age and fetal sex chromosome aneuploidies: a 8-year single institution experience in China

**DOI:** 10.1186/s13039-021-00545-2

**Published:** 2021-05-10

**Authors:** Hongge Li, Yuchan Mao, Jinglei Jin

**Affiliations:** Women’s Hospital, School of Medicine, Zhejiang University, 1, Xueshi Road, Hangzhou, Zhejiang 310006 People’s Republic of China

**Keywords:** Advanced maternal age (AMA), Sex chromosome aneuploidies (SCAs), Second-trimester amniocentesis, Prenatal diagnosis, Cytogenetic diagnosis

## Abstract

**Background:**

Although a few studies have investigated a possible association between maternal age and fetal sex chromosome aneuploidies (SCAs), most of these studies were limited to advanced maternal age (AMA) women and the results were conflicting. This study aimed to investigate the correlation between maternal age and common fetal SCAs (including 45,X, 47,XXY, 47,XXX and 47,XYY) in pregnant women of different ages that not only limited to AMA women. We retrospectively investigated a 8-year experience of prenatal diagnosis for fetal chromosome aberrations by second-trimester amniocentesis at a university teaching hospital in China. 20,409 amniotic fluid specimens collected at 19–22^+6^ gestational weeks were included in this study. The women were categorized into five age groups (≤ 23, 24–28, 29–33, 34–38, 39^+^ years) based on maternal age at the time of amniocentesis and entered as a categorical variable in all samples. The correlation between fetal SCAs and maternal age was determined using the logistic regression analysis. A chi-square test was performed to compare the incidence of fetal SCAs among age groups.

**Results:**

A total of 179 cases of fetal SCAs were detected, and the incidence was 8.77‰ (about 1/114). The incidence of fetal SCAs increased significantly with advancing maternal age (SE, 0.014; odds ratio, 1.044; *P* = 0.002). Specifically, the incidence of 45,X (SE, 0.037; odds ratio, 0.916; *P* = 0.017) and 47,XXY (SE, 0.024; odds ratio, 1.127; *P* = 0.000) had significant correlation with maternal age, while the incidence of 47,XXX and 47,XYY had no correlation with maternal age (*P* = 0.473; *P* = 0.272, respectively). The incidence of fetal SCAs was also significantly different among age groups (*χ*^2^ = 10.197, *P* = 0.037 < 0.05), from 5.81 per 1000 fetuses at the 24–28 years to 10.92 per 1000 at the 39^+^ years.

**Conclusions:**

Maternal age was ascertained to be a strong risk factor for fetal SCAs.

## Background

Chromosomal abnormalities are the leading causes of stillbirth and neonatal birth defects [1]. The most common numerical chromosomal abnormalities observed in the liveborns are Down syndrome (trisomy 21 or T21), Edwards syndrome (trisomy 18 or T18), Patau syndrome (trisomy 13 or T13) and sex chromosome aneuploidies (SCAs) [2]. SCAs occur with a frequency of 1 in 500, an incidence greater than that of trisomy 21 [3]. SCAs are a common group of chromosome disorders characterized by the loss or gain of one or more sex chromosomes, including 45,X (Turner syndrome; 1/2000 female livebirths), 47,XXX (Triple X syndrome; 1/1000 female livebirths), 47,XXY (Klinefelter syndrome; 1/600 male livebirths), 47,XYY (47,XYY syndrome; 1/1000 male livebirths), as well as rare SCAs such as 48,XXXX, 48,XXXY, 48,XXYY and 69,XXX [4, 5]. The karyotype analysis of amniotic fluid cells in the second-trimester is the main method for detecting fetal chromosomal aberrations and is regarded as the gold standard for cytogenetic diagnosis currently [6].

A number of risk factors can increase the probability of chromosomal abnormalities during pregnancy. Since the 1980s, it is clearly shown that maternal age could be the most important risk factor for fetal chromosomal abnormalities [7]. To date, it is widely accepted that the risk for autosomal aneuploidies (including T21, T18 and T13) is directly correlated with maternal age [8]. Even though a few studies have investigated a possible association between maternal age and fetal SCAs including 45,X, 47,XXY, 47,XXX and 47,XYY, most of these studies were limited to indication of AMA and the results were not conclusive. The study of the correlation between maternal age and fetal SCAs (including 45,X, 47,XXY, 47,XXX and 47,XYY) from previously published studies is shown in Table 1. In summary, several large-scale epidemiological studies on the relationship between maternal age and chromosomal abnormalities were multicenter collaborative studies, and almost all of these studies were limited to AMA women and these conclusions were conflicting.

**Table 1:** The correlation study between maternal age and fetal SCAs (including 45,X, 47,XXY, 47,XXX and 47,XYY) from previously published studies

Diagnostic center	Number of pregnancies tested	Number with maternal age		Maternal age specific rates (%) for SCAs				References
		≥ 35 years	< 35 years	45,X	47,XXY	47,XXX	47,XYY	
Europe and outside Europe	52,965	52,965	0	Negative correlation	Relevant	Relevant	Irrelevance	[9]
China	46,258	46,258	0	Irrelevance	Relevant	Relevant	Irrelevance	[10]
Italy	88,965	51,758	37,207	Borderline significance	Relevant	Relevant	Borderline significance	[11]
South Korea	15,381	15,381	0	Relevant	Relevant	Relevant	–	[12]

Maternal age was recorded at amniocentesis, except for that by Kim et al. [12], in which maternal age was recorded at excepted date of delivery. In each of the studies, amninocentesis was performed on the sole indication of maternal age

The intention of this study was to investigate the correlation between maternal age and common fetal SCAs (including 45,X, 47,XXY, 47,XXX and 47,XYY). Here, we retrospectively investigated a single center experience of prenatal diagnosis for fetal chromosomal aberrations during the last 8 years (from January 1st 2011, until December 31st 2018) in the Women's Hospital of Zhejiang University, a university teaching hospital, in southern China. Unlike previous publications, this study compared the incidence of fetal SCAs in pregnant women of different ages that were not limited to AMA women, and this study did not exclude pregnant women with family history of chromosomal abnormalities and fetal abnormalities detected by ultrasound or other indications of an unfavorable prenatal diagnosis, which could better reflect the maternal age-related risk of fetal SCAs.

## Materials and methods

### Subjects

This was a retrospective cohort study of all consecutive singleton pregnancies who underwent second-trimester amniocentesis between January 1st 2011 and December 31st 2018 at the Women's Hospital of Zhejiang University. Amniocentesis and karyotype analysis was performed in 20,672 pregnant women during the last 8 years. Among these, 253 cases were excluded for multiple gestations and 10 cases were excluded for failing in amniotic fluid culture. Eventually, 20,409 singleton amniotic fluid specimens were eligible for the study. Age of these women ranged from 18 to 52 years, with an average age of 33.09 years (± 5.447). All pregnant women underwent amniocentesis at the 19–22^+6^ weeks of gestation. Gestational week was determined based on the date of the last menstrual period (LMP) or ultrasonic examination (biparietal diameter) if the value differed from the LMP-derived gestational week by ≥ 3 days. The indications for amniocentesis have strictly followed the guideline of Chinese government, including AMA (35 years or older at the expected date of delivery), positive results of maternal serological screening (MSS) (T21 ≥ 1/270, T18 ≥ 1/350), abnormal ultrasound findings (including increased nuchal translucency before the first-trimester screening, fetal structural abnormalities, abnormal ultrasound soft markers), positive results from noninvasive prenatal testing (NIPT), family history of chromosomal abnormalities (a previous child with chromosomal abnormalities or paternal/maternal carrying chromosomal abnormalities, including sex chromosomal abnormalities), adverse pregnancy history (a history of intrauterine fetal death or aborted fetuses), intracytoplasmic sperm injection (ICSI) or in vitro fertilization embryo transfer (IVF-ET), parental anxiety and others. In the study, maternal age was used as the only indication for statistical analysis, regardless of the main referral indications for amniocentesis. Traditionally, prenatal diagnosis has been offered to women aged 35 years or older at the expected date of delivery. Since it was found to be impractical to collect maternal age corrected for the expected delivery date, this study was based on the ages given at the time of amniocentesis, not the expected date of delivery. Most of 34-year-old pregnant women would give birth at the age of 35-year old, so we classified the 34-year-old women to the advanced age group. The cases were categorized into the following 5 age groups at intervals of 5 years according to maternal age (≤ 23, 24–28, 29–33, 34–38, 39^+^ years) and entered as a categorical variable in all samples. The patients in only 270 cases were ≥ 44 years of age; they were included in the 39–43-year-old group (39^+^ years). In this study, we calculated the incidence of common fetal SCAs (45,X; 47,XXX; 47,XXY; 47,XYY), excluding rare fetal SCAs such as 48,XXXX, 48,XXXY, 48,XXYY and 69,XXX. This study was approved by the Scientific Research Ethics Committee of the Women's Hospital of Zhejiang University. This was a retrospective study of the clinical database with no intervention and no informed consent was required.

### Karyotyping

Conventional cytogenetic analysis was performed on all samples according to the standard protocol of the Women's Hospital of Zhejiang University of Human Cytogenetics Guidelines, as previously described [13]. Amniotic fluid was centrifuged and cultured immediately after the specimen was obtained. 20 mL amniotic fluid (with the first 1–2 mL amniotic fluid was discarded) was divided into two sterile centrifuge tubes for centrifugation at 1500 rpm for 10 min, and the supernatant was discarded. The cell suspension was inoculated into culture bottle (Corning, Falcon®, catalog number: 353108) containing of 5 mL amniotic fluid cell medium (BIO-AMF-2., Biological Industries Ltd, Kibbutz Beit-Haemek, Israel) in 5% CO_2_ incubator at 37 °C for 6–7 days, then the medium was changed. The cell growth was observed every day after the medium was changed. When the amniotic fluid cells adhered to the wall and exhibited multiple clones under an inverted microscope, the amniotic fluid cells in each culture bottle were collected separately. Metaphase chromosomes with targeted 400-band level were obtained by making sections and Giemsa banding. Cytogenetic analysis was performed by Leica GLS120 Automated Nuclear Scanning System (CytoVision., Leica, Wetzlar, German) after G-banding, and at least 30 metaphases were counted and 5 metaphases were analyzed for each patient. If there were different cell lineages in the same patient, the count was increased to 50–100 metaphases to establish the mosaicism. Karyotype results were described by certified physicians following the criteria established by the International System for Human Cytogenetic Nomenclature guidelines (ISCN, 2016, 5th edition) [14].

### Statistical analysis

The data used for analysis did not contain identifiable personal information to protect individuals’ privacy. Data were presented as mean ± SD and *n* (% or ‰). Logistic regression analysis was performed to analyze the correlation between maternal age and fetal SCAs. Chi-square test was performed to compare the incidence of fetal SCAs among age groups. Fisher's exact test was used when there was a frequency count < 5. *P* < 0.05 was considered statistically significant. Moreover, we calculated the odds ratio (OR) and confidence interval (CI) at 95% CI. SPSS version 16.0 (SPSS Inc., Chicago, IL, USA) was used for the statistical analysis.

## Results

### Clinical characteristics of the entire cohort in the study

**Fig. 1:**
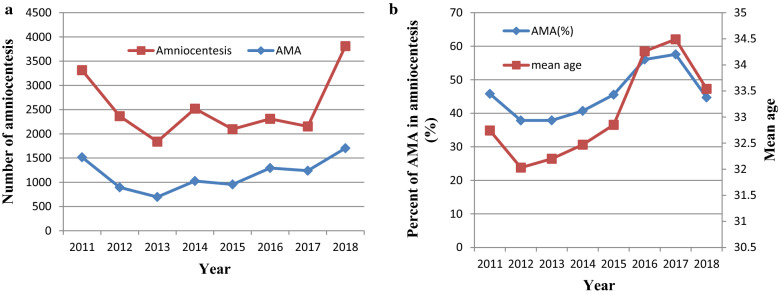
(**a**) Change in the relative number of amniocentesis and women with advanced maternal age (≥ 35) undergoing amniocentesis at the Women's Hospital of Zhejiang University, 2011–2018. (**b**) Trend in the proportion of women with advanced maternal age undergoing amniocentesis at the Women's Hospital of Zhejiang University, and mean age of these women, 2011–2018

**Fig. 2:**
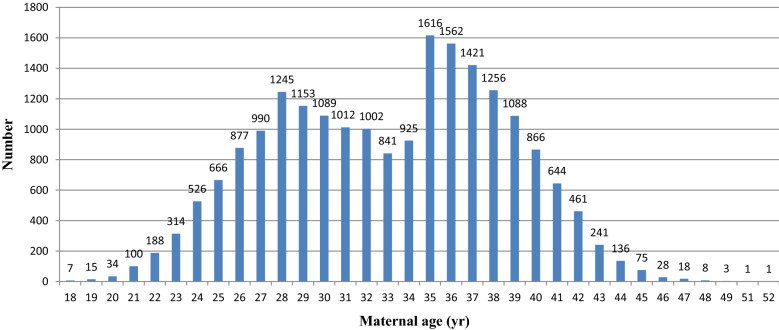
Maternal age distribution in the entire study cohort

This study included 20,409 singleton pregnancies and 186 cases of fetal SCAs were diagnosed. The clinical significance of fetal SCAs (by year) in the entire cohort is shown in Table 2. Excluding 7 rare fetal SCAs (including 1 case of 48,XXXY, 2 cases of 48,XXXX, 1 case of 48,XXYY and 3 cases of 69,XXX), 179 cases of common fetal SCAs were diagnosed with an incidence rate of 8.77‰ (about 1/114). The most common fetal SCAs was 47,XXY in 70 cases (39.11%), followed by 47,XXX in 44 (24.58%), 47,XYY in 39 (21.79%) and 45,X in 26 (14.53%). The proportion of AMA women receiving amniocentesis accounted for about half (45.71%, 9329/20,409) of the clinical samples. Relative number of amniocentesis per year and correlated with maternal age are presented in Fig. 1. The results showed that both, proportion of AMA women and the mean age of the women increased between 2012 and 2017 and decreased later. Maternal age distribution of the entire study cohort and the incidence (per 1000) of fetal SCAs by maternal age are shown in Fig. 2 and Table 3. Two birth peaks appeared at 28 and 35 years old. The incidence of 47,XXY, 47,XXX and 47,XYY was found to increase dramatically at the age of 44. In addition, only 76 (0.37%, 76/20,409) were diagnosed as having chromosome mosaicism in this study, shown in Table 4.

**Table 2:** Clinical significance of fetal SCAs (by year) in the entire cohort

Year	No. of patients	AMA	% of AMA (%)	Mean age ± SD	Sex chromosome aneuploidies								
					Total	45,X	47,XXY	47,XXX	47,XYY	48,XXXY	48,XXXX	48,XXYY	69,XXX
2011	3314	1518	45.81	32.74 ± 5.359	9	2	1	2	2	1			1
2012	2364	895	37.86	32.03 ± 5.430	10	3	3	1	2				1
2013	1840	697	37.88	32.20 ± 5.476	16	4	7	2	2		1		
2014	2520	1026	40.71	32.47 ± 5.282	13	4	2	4	3				
2015	2097	955	45.54	32.85 ± 5.347	14	1	6	3	4				
2016	2309	1294	56.04	34.26 ± 5.143	32	3	13	8	7			1	
2017	2154	1240	57.57	34.49 ± 5.456	22	1	10	5	4		1		1
2018	3811	1704	44.71	33.54 ± 5.530	70	8	28	19	15				
Total	20,409	9329	45.71	33.09 ± 5.447	186	26	70	44	39	1	2	1	3

**Table 3:** The incidence (per 1000) of 45,X, 47,XXY, 47,XXX and 47,XYY by maternal age in the study

Maternal age	No. of patients	45,X	47,XXY	47,XXX	47,XYY	All SCAs
18	7	0 (0)	0 (0)	0 (0)	0 (0)	0 (0)
19	15	0 (0)	0 (0)	0 (0)	0 (0)	0 (0)
20	34	0 (0)	0 (0)	0 (0)	0 (0)	0 (0)
21	100	0 (0)	0 (0)	0 (0)	0 (0)	0 (0)
22	188	0 (0)	1 (5.32)	1 (5.32)	0 (0)	2 (10.64)
23	314	1 (3.18)	0 (0)	1 (3.18)	0 (0)	2 (6.37)
24	526	0 (0)	1 (1.90)	1 (1.90)	0 (0)	2 (3.80)
25	666	0 (0)	1 (1.50)	2 (3.00)	1 (1.50)	4 (6.01)
26	877	2 (2.28)	0 (0)	2 (2.28)	2 (2.28)	6 (6.84)
27	990	5 (5.05)	1 (1.01)	0 (0)	1 (1.01)	7 (7.07)
28	1245	1 (0.80)	1 (0.80)	3 (2.41)	1 (0.80)	6 (4.82)
29	1153	5 (4.34)	2 (1.73)	2 (1.73)	1 (0.87)	10 (8.67)
30	1089	2 (1.84)	1 (0.92)	3 (2.75)	0 (0)	6 (5.51)
31	1012	0 (0)	2 (1.98)	2 (1.98)	3 (2.96)	7 (6.92)
32	1002	4 (3.99)	1 (1.00)	1 (1.00)	6 (5.99)	12 (11.98)
33	841	1 (1.19)	2 (2.38)	0 (0)	1 (1.19)	4 (4.76)
34	925	1 (1.08)	4 (4.32)	2 (2.16)	5 (5.41)	12 (12.97)
35	1616	1 (0.62)	7 (4.33)	7 (4.33)	4 (2.48)	19 (11.76)
36	1562	0 (0)	11 (7.04)	3 (1.92)	2 (1.28)	16 (10.24)
37	1421	0 (0)	6 (4.22)	2 (2.11)	4 (4.22)	12 (8.44)
38	1256	1 (0.80)	5 (3.98)	4 (3.18)	3 (2.39)	13 (10.35)
39	1088	1 (0.92)	7 (6.43)	0 (0)	1 (0.92)	9 (8.27)
40	866	0 (0)	5 (5.77)	2 (2.31)	2 (2.31)	9 (10.39)
41	644	0 (0)	5 (7.76)	1 (1.55)	0 (0)	6 (9.32)
42	461	0 (0)	3 (6.51)	1 (2.17)	1 (2.17)	5 (10.85)
43	241	1 (4.15)	1 (4.15)	1 (4.15)	0 (0)	3 (12.45)
44	136	0 (0)	2 (14.71)	3 (22.06)	1 (7.35)	6 (44.12)
45	75	0 (0)	0 (0)	0 (0)	0 (0)	0 (0)
46	28	0 (0)	1(35.71)	0 (0)	0 (0)	1 (35.71)
47	18	0 (0)	0 (0)	0 (0)	0 (0)	0 (0)
48	8	0 (0)	0 (0)	0 (0)	0 (0)	0 (0)
49	3	0 (0)	0 (0)	0 (0)	0 (0)	0 (0)
51	1	0 (0)	0 (0)	0 (0)	0 (0)	0 (0)
52	1	0 (0)	0 (0)	0 (0)	0 (0)	0 (0)
Total	20,409	26 (1.27)	70 (3.42)	44 (2.16)	39 (1.91)	179 (8.77)

**Table 4:** The characteristics of 76 cases diagnosed with sex chromosome mosaicism in this study

Case	Sex chromosome mosaicisms	n	Case	Sex chromosome mosaicisms	**n**
Case 1	mos 45,X[13]/46,XX[67]	1	Case 39	mos 46,XX[40]/45,X[9]/45,X, + mar[8]	1
Case 2	mos 45,X[28]/46,XY[2]	1	Case 40	mos 46,XX[44]/45,(X)[5]/47,XXX[1]	1
Case 3	mos 45,X[29]/46,XX[41]	1	Case 41	mos 46,XX[68]/45,X[15]	1
Case 4	mos 45,X[48]/46,X,i(X) (q10)[2]	1	Case 42	mos 46,XX[99]/45,X[1]	1
Case 5	mos 45,X[9]/46,XX[41]	1	Case 43	mos 46,XX[2]/46,XY[98]	1
Case 6	mos 45,X,der(5)[3]/46,XX,der(5)[57]	1	Case 44	mos 46,XX[23]/45,X[7]	1
Case 7	mos 45,X,idic(Y)[76]/45,X[24]	1	Case 45	mos 46,XX[39]/45,X[11]	1
Case 8	mos 45,X,inv(9)[32]/46,XX,inv(9)[18]	1	Case 46	mos 46,XX[40]/47,XXX[10]	1
case 9	mos 45,X[17]/46,XX[33]	1	Case 47	mos 46,XX[40]/47,XYY[10]	1
Case 10	mos 45,X[18]/46,X,i(Xq)[12]	1	Case 48	mos 46,XX[42]/45,X[8]	1
Case 11	mos 45,X[18]/46,XX[82]	1	Case 49	mos 46,XX[46]/45,X[4]	1
Case 12	mos 45,X[19]/46,XX[11]	1	Case 50	mos 46,XX[48]/47,XXX[2]	1
Case 13	mos 45,X[2]/46,XX[98]	1	Case 51	mos 46,XX[50]/46,XX,der(13)[2]	1
Case 14	mos 45,X[2]/46,XY,der(5)[2]/47,XY, + 2,der(15)[1]	1	Case 52	mos 46,XX[54]/45,X[7]	1
Case 15	mos 45,X[2]/46,XY[120]	1	Case 53	mos 46,XY[27]/45,X[37]	1
Case 16	mos 45,X[2]/46,XY[48]	1	Case 54	mos 46,XY,del(Y)[30]/45,X[21]	1
Case 17	mos 45,X[21]/46,XY[12]	1	Case 55	mos 46,XY[25]/45,X[5]	1
Case 18	mos 45,X[23]/46,XX[52]	1	Case 56	mos 46,XY[26]/45,X[4]	1
Case 19	mos 45,X[25]/47,XXX[3]/46,XX[1]	1	Case 57	mos 46,XY[43]/46,XX[2]	1
Case 20	mos 45,X[27]/46,X,der(X) (p10)[23]	1	Case 58	mos 46,XY[46]/45,X[4]	1
Case 21	mos 45,X[29]/46,XX[21]	1	Case 59	mos 46,XY[46]/45,X[4]	1
Case 22	mos 45,X[3]/46,XY[47]	1	Case 60	mos 46,XY[48]/45,X[2]	1
Case 23	mos 45,X[3]/46,XY[47]	1	Case 61	mos 46,XY[58]/46,XX[2]	1
Case 24	mos 45,X[3]/46,XY[47]	1	Case 62	mos 46,XY[78]/46,XX[22]	1
Case 25	mos 45,X[30]/46,Xi(Yq)[20]	1	Case 63	mos 47,XXX[2]/46,XX[48]	1
Case 26	mos 45,X[39]/46,XY[3]/47,XYY[4]	1	Case 64	mos 47,XXX[40]/46,XX[10]	1
Case 27	mos 45,X[47]/46,X,i(X)(q10)[3]	1	Case 65	mos 47,XXX[84]/45,X[16]	1
Case 28	mos 45,X[47]/47,XXX[3]	1	Case 66	mos 47,XXX[2]/46,XX[48]	1
Case 29	mos 45,X[5]/46,XX[25]	1	Case 67	mos 47,XXX[46]/46,XX[4]	1
Case 30	mos 45,X[5]/46,XX[75]	1	Case 68	mos 47,XXY[22]/46,XY[11]	1
Case 31	mos 45,X[7]/46,XX[43]	1	Case 69	mos 47,XXY[31]/46,XY[19]	1
Case 32	mos 45,X[7]/46,XX[93]	1	Case 70	mos 47,XXY[40]/46,XX[12]	1
Case 33	mos 45,X[8]/46,XX[42]	1	Case 71	mos 48,XXYY[28]/47,XXY[22]	1
Case 34	45,X[9]/46,XX[21]	1	Case 72	mos 47,XYY[38]/45,X[9]/47,XY, + 17[3]	1
Case 35	45,X[9]/46,XX[41]	1	Case 73	mos 47,XYY[6]/46,XY[60]	1
Case 36	45,X[10]/46,XX[40]	1	Case 74	mos 46,X,der(Y)[29]/45,X[23]	1
Case 37	45,X[6]/46,XX[44]	1	Case 75	mos 46,X,r(X)[15]/45,X[15]	1
Case 38	45,X[7]/46,XX[43]	1	Case 76	mos 46,X,der(Y)[22]/45,X[8]	1

### The incidence of fetal SCAs depended on clinical indications

In the study, AMA and positive results of MSS were the most frequent reasons for referral, accounting for 47.69% and 34.65%, respectively. The most of fetal SCAs were detected with the indication of AMA (111/179, 62.01%), followed by abnormal NIPT results (34/179, 19.00%), abnormal ultrasound findings (16/179, 8.94%), positive result of MSS (15/179, 8.38%) and others (3/179, 1.68%). However, the incidence of SCAs varied by indications: 83.74‰ for abnormal NIPT results, 17.98‰ for abnormal ultrasound findings, 11.40‰ for AMA, 2.12‰ for positive results of MSS and 1.30‰ for others. AMA was the most frequent referral indication for 47,XXY, 47,XXX and 47,XYY, while abnormal ultrasound findings were the most frequent referral indication for 45,X. Three pregnant women with the indication of others were found to have fetal SCAs, including 1 case of monogenetic disease, 1 case of a previous child with 5p-, and 1 case of a previous child with 21 trisomy. None of the pregnant women with fetal SCAs were found to have the referral indication of paternal/maternal carrying sex chromosome abnormalities or a family history of a previous child with sex chromosome abnormalities. The incidence and proportion of fetal SCAs by referral indications are showed in Table 5.

**Table 5:** The frequency and proportion of fetal SCAs by referral indications

Referral indications	No. of patients (%)	SCAs (%)	SCAs (‰) by indications	SCAs			
				45,X	47,XXY	47,XXX	47,XYY
AMA	9734 (47.69%)	111 (62.01%)	11.40‰	5 (19.23%)	57(81.43%)	26(59.09%)	23(58.97%)
Positive results of MSS	7072 (34.65%)	15 (8.38%)	2.12‰	5 (19.23%)	1(1.43%)	3(6.82%)	6(15.38%)
Abnormal ultrasound findings	890 (4.36%)	16 (8.94%)	17.98‰	10 (38.46%)	2(2.86%)	3(6.82%)	1(2.56%)
Abnormal NIPT results	406 (1.99%)	34 (19.00%)	83.74‰	6 (23.08%)	9(12.86%)	12(27.27%)	7(17.95%)
Others	2307 (11.30%)	3 (1.68%)	1.30‰	0 (0.00%)	1(1.43%)	0(0.00%)	2(5.13%)
Total	20,409 (100.00%)	179 (100.00%)	8.77‰	26 (14.53%)	70 (39.11%)	44(24.58%)	39(21.79%)

### The correlations between maternal age and the incidence of fetal SCAs

**Fig. 3:**
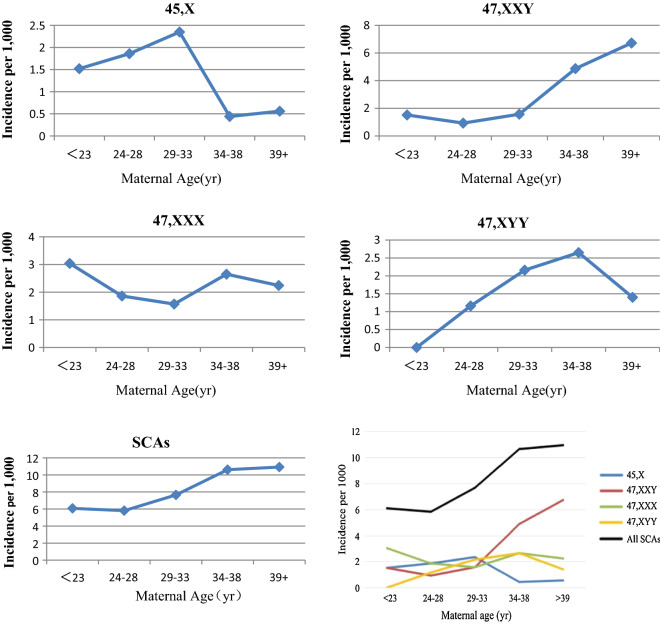
Trend in the incidence (per 1000) of fetal SCAs based on maternal age groups

Logistic regression analysis was performed to analyze the correlation between maternal age and the incidence of fetal SCAs, showed in Table 6. According to the results of logistic regression analysis, the incidence of fetal SCAs was significantly associated with maternal age (*P* = 0.002), and the odds ratio tended to increase by 1.044 times as maternal age increased by one year. The incidence of 45,X and 47,XXY showed significant correlation with maternal age (*P* = 0.017; *P* = 0.000, respectively), and the odds ratio of 45,X tended to increase by 0.916 times and the odds ratio of 47,XXY tended to increase by 1.127 times as maternal age increased by one year. However, the incidence of 47,XXX and 47,XYY was not found to be significantly correlated with maternal age (*P* = 0.473; *P* = 0.272, respectively). Trends in the incidence of fetal SCAs based on maternal age groups are shown in Fig. 3. The incidence of 47,XXY and all SCAs was basically positively correlated with maternal age, respectively, however, a significant inverse relationship with maternal age was found for 45,X at the upper end of the 29–33 age range. Unlike the incidence of 47,XXX being completely independent of maternal age, the incidence of 47,XYY before the 34–38 years was completely dependent on the maternal age, and then decreased sharply.

**Table 6:** Regression coefficient and standard errors of logistic regression equations for fetal SCAs diagnosed in the study

	Constant term		Maternal term				
	A	Standard error	B	Standard error	Odds ratio	95% CI	*P* value
45,X	− 3.861	1.143	− 0.088	0.037	0.916	0.852–0.984	0.017 < 0.05
47,XXY	− 9.817	0.894	0.119	0.024	1.127	1.074–1.182	0.000 < 0.05
47,XXX	− 6.807	0.953	0.020	0.028	1.020	0.966–1.078	0.473 > 0.05
47,XYY	− 7.360	1.030	0.033	0.030	1.033	0.975–1.096	0.272 > 0.05
All SCA_S_	− 6.186	0.490	0.043	0.014	1.044	1.016–1.073	0.002 < 0.05

### Comparison of the incidence of SCAs among different age groups

A chi-square test was performed to compare the incidence of fetal SCAs among different age groups; the statistical results are shown in Table 7. The incidence of fetal SCAs was significantly different among groups (*χ*^2^ = 10.197, *P* = 0.037), but there were no significant differences between adjacent groups (*P* > 0.05). The incidence of 45,X and 47,XXY was also significantly different among groups (*χ*^2^ = 10.977, *P* = 0.027; *χ*^2^ = 29.159, *P* = 0.000, respectively), while the incidence of 47,XXX and 47,XYY was not found to be significantly different among groups (*χ*^2^ = 2.027, *P* = 0.731; *χ*^2^ = 5.145, *P* = 0.273, respectively). We further compared the incidence of 45,X and 47,XXY between adjacent groups, and found that the incidence of 45,X and 47,XXY was significantly different only between the 29–33 years and the 34–38 years (*P* = 0.004; *P* = 0.002, respectively) (Table 8). However, the incidence of 45,X in the 34–38 years (0.44‰, 1:2272) was lower than the 29–33 years (2.35‰, 1:426), while the incidence of 47,XXY was the opposite (4.87‰, 1:205 vs. 1.57‰, 1:637).

**Table 7:** Comparison of the incidence of fetal SCAs among different age groups

Age groups	No. of patients	45,X^a^	47,XXY^b^	47,XXX^c^	47,XYY^d^	All SCA_s_^e^
≤ 23	658	1(1.52)	1(1.52)	2(3.04)	0(0)	4(6.08)
24–28	4304	8(1.86)	4(0.93)	8(1.86)	5(1.16)	25(5.81)
29–33	5097	12(2.35)	8(1.57)	8(1.57)	11(2.16)	39(7.65)
34–38	6780	3(0.44)	33(4.87)	18(2.65)	18(2.65)	72(10.62)
39^+^	3570	2(0.56)	24(6.72)	8(2.24)	5(1.40)	39(10.92)
Total	20,409	26(1.27)	70(3.43)	44(2.16)	39(1.91)	179(8.77)

Data are presented as n (per 1000), unless otherwise indicated

^a^The incidence of 45,X was significantly different among the groups (*χ*^2^ = 10.977, *P* = 0.027 < 0.05)

^b^The incidence of 47,XXY was significantly different among the groups (*χ*^2^ = 29.159, *P* = 0.000 < 0.05)

^c^The incidence of 47,XXX was significantly different among the groups (*χ*^2^ = 2.027, *P* = 0.731 > 0.05)

^d^The incidence of 47,XYY was significantly different among the groups (*χ*^2^ = 5.145, *P* = 0.273 > 0.05)

^e^The incidence of all SCAs was significantly different among the groups (*χ*^2^ = 10.197, *P* = 0.037 < 0.05)

**Table 8:** Comparison of the incidence of 45,X and 47,XXY between the 29–33 years and the 34–38 years

Age groups	No. of patients	45,X				47,XXY			
		Number/incidence	PPV	χ^2^	*P*	Number/Incidence	PPV	*χ*^2^	*P*
29–33	5097	12 (2.35)	1:426	8.432	0.004	8 (1.57)	1:637	9.198	0.002
34–38	6780	3 (0.44)	1:2272			33 (4.87)	1:205		

Data are presented as *n* (per 1000), unless otherwise indicated

*PPV* Positive predictive value

## Discussion

The present study reported one the largest cohorts of invasive prenatal diagnosis by amniotic fluid specimens from a representative database of a single cytogenetics laboratory in China. An advantage of the study was that the data was collected from the same prenatal diagnosis center, thus avoiding bias in the medical performance, and it was from a teaching hospital of well-known universities with a case ascertainment of approximately 100% with high data quality. In addition, the level of misdiagnosis could be determined by strictly following the national guideline and follow-up system. In the study, the incidence of all SCAs (8.77‰, about 1/114), 45,X (1.27‰, 1/787), 47,XXY (3.42‰, 1/292), 47,XXX (2.16‰, 1/463), 47,XYY (1.91‰, 1/524) was much higher than earlier publication [4, 5]. There were several factors that affect this result. The most important reason might be that the current knowledge about the incidence of chromosomal abnormalities in the general population came from studies in newborns carried out in the 1970s, this data has not been updated and the earlier studies had technical and methodological limitations. This is a different incidence rate than the earlier referenced incidence rate that was reported in terms of all live births.

In this study, 179 cases of fetal SCAs and 76 cases of sex chromosome mosaicism were diagnosed. The karyotype analysis of umbilical cord blood was recommended in cases of chromosome mosaicism was diagnosed through amniocentesis, and the umbilical cord blood karyotype results were also consistent with the prenatal amniocentesis results. The follow-up investigation results from our hospital prenatal diagnostic center showed that most of the fetuses with SCAs, including sex chromosome mosaicisms, have implemented induction of abortion, which was an important cause for the higher incidence of fetal SCAs in the study. Second, amniocentesis was only performed on women who were already marked as higher risk. Especially, as the largest prenatal diagnosis center and the main referral centers in Zhejiang Province, many patients with the referral indications of amniocentesis were likely to be referred to the hospital for further evaluation and management. And this study did not exclude pregnant women with family history of chromosomal abnormalities, ultrasound abnormalities, adverse pregnancy history and other indicators, which might increase the proportion of chromosomal abnormalities. Moreover, new screening technologies as alternatives had been used to detect chromosomal abnormalities, especially, the increasing reliability of NIPT has facilitated far more first trimester screening for the major aneuploidies [15]. NIPT used techniques based on genome-wide massively parallel sequencing (MPS) (also called as next-generation sequencing, NGS) to analyze cell-free fetal DNA isolated from maternal plasma. NIPT as a successful application in routine clinical practice has been widely used to detect trisomy 21, 18, 13 and SCAs [16]. In the present study, it was found that 19.00% of pregnant women with fetal SCAs had the indication of abnormal NIPT results, and the indication had the highest positive predictive value for SCAs (83.74‰). It must be noted that NIPT has only been available in China since 2012, and given the length of the study period, this might have a significant impact on the results. The discrepancy might also be related to the age distribution of the participants since the frequency of some chromosomal abnormalities was directly associated with maternal age [17]. In this study, the percentage of AMA women has gradually increased since 2012, especially in 2016 and 2017 reaching 56.04% and 57.57%, respectively, which might be due to the implementation of the “second-child policy” in China. Therefore, it was reasonable to have a higher incidence rate of fetus SCAs.

The incidence of fetal SCAs depends on clinical indications [18]. AMA was the most frequent referral indication for 47,XXY, 47,XXX and 47,XYY, while abnormal ultrasound findings was the most frequent referral indication for 45,X, which was consistent with the previous study [19]. This study indicated that the incidence of fetal SCAs was significantly related to maternal age, ranging from the lowest of 5.81 per 1000 fetuses at the 24–28 years to the highest of 10.92 per 1000 fetuses at the 39^+^ years. The incidence of fetal SCAs was significantly different among groups, but not significantly different between adjacent groups. This study also found that the incidence of 45,X and 47,XXY was significantly related to maternal age, while the incidence of 47,XXX and 47,XYY was not related to maternal age. And the incidence of 45,X and 47,XXY was significantly different only between the 29–33 years and the 34–38 years. Specifically, unlike the incidence of 47,XXY, which gradually increased with advancing maternal age, the incidence of 45,X reached the peak of 2.35‰ (1: 426) at the 29–33 years, and then decreased sharply to 0.44‰ (1: 2272) at the 34–38 years. However, because the number of research subjects for each type of abnormality was too small, we could not tell at what age the cutoff occured.

Turner’s syndrome is the only complete monosomy that is viable in human beings. Some studies have shown that the loss of the X chromosome in the peripheral blood lymphocytes increased with maternal age [20–22], but clinical studies have shown that young women have a higher incidence of 45,X [23]. However, the incidence of 45,X increased with maternal age and reached the peak at a specific maternal age, then tended to decline at the upper limit of the age range, which was consistent with as Ferguson-Smith et al. described [9]. Does this mean that when the age of pregnant women reaches a certain threshold, the incidence of 45,X will decline? Does this happen by accident? Uematsu A [24] found that 45,X is not related to advanced maternal age and it is more likely due to instability of the Y chromosome since 75–80% of X chromosomes in patients with 45,X are maternal in origin. The study failed to rule out confounding factors such as father's age, which required expanding the sample size to seek possible influencing factors in the further studies.

47,XXY is the most frequent genetic disorder and accounts for approximately two-thirds of all the cytogenetic abnormalities [25]. In the study, 47,XXY was the most common SCAs and accounted for 39.11% of all SCAs. Similar to previous studies [9–11], we found that 47,XXY showed significant correlation with maternal age. The incidence of 47,XXY was basically positively correlated with maternal age, except for the ≤ 23 years, which might be due to the number of patients and affected fetuses was very small in size. According to the results of this study, the incidences of 47,XXX and 47,XYY were not found to be significantly correlated with maternal age. However, trend in the incidence of fetal SCAs showed that the incidence of 47,XXX was completely independent of maternal age, while the incidence of 47,XYY was completely dependent on the maternal age before the 34–38 years and then decreased sharply at the 39^+^ years, this necessitates further studies on larger cohorts in the future. In addition, the incidence of 47,XXY, 47,XXX and 47,XYY was found to increase dramatically at the age of 44. However, because the number of cases at the age of 44 or older was very small, analysis of the correlation between fetal chromosomal abnormalities and extreme maternal age was not possible.

Aneuploidies are caused by nondisjunction or abnormal segregation of chromosomes during the meiotic cellular division process. The most common cause of SCAs is nondisjunction, which can occur during meiosis or the early stages of postzygotic development. Premature centromere division indicated a dysfunction of the X-chromosome centromere with aging, and this dysfunction was the basic cause of age-related aneuploidy. The incidence of SCAs in human lymphocytes increased with AMA have been noted for several decades [21, 26]. This study supports the conclusion that maternal age was an important risk factor for fetal SCA. This study aimed to investigate maternal age-specific rates for common fetal SCAs (including 45,X, 47,XXY, 47,XXX and 47,XYY) in pregnant women of different ages that not only limited to AMA women. In this study, the incidence of 45,X and 47,XXY had significant correlation with maternal age, while the incidence of 47,XXX and 47,XYY had no correlation with maternal age. Although previous studies have investigated the association between maternal age and fetal SCAs**,** most of these studies were limited to AMA women and the results were conflicting [9–12]. This might caused by many confounding factors, among which father’s age was an important factor. Nondisjunction events resulting in a loss of a paternal sex chromosome are the most common genetic mechanism that leads to 45,X monosomy (about 70–80%) and more than half of 47,XXY karyotypes result from paternal errors at meiosis I, with the rest from maternal meiosis I or II, or postzygotic mitotic errors, whlie 47,XYY can arise only from paternal errors, either at meiosis II (about 85%) or from postzygotic events [4]. Published work on other SCAs is scarce. In this study, due to the incompleteness of the data, this study failed to rule out confounding factors such as maternal parity history and father's age, however, previous studies have found that fetal chromosomal aneuploidies might be related to these factors [27, 28]. Our studies in the future will focus on the influence of paternal age on sex chromosomes.

## Conclusion

Maternal age was ascertained to be a strong risk factor for fetal SCAs, and the incidence of fetal SCAs depended on clinical indications. This study compared the incidence of common fetal SCAs in pregnant women of different ages throughout the child-bearing period that not only limited to AMA women, which could better reflect the relationship between fetal SCAs and maternal age.

## Data Availability

The datasets generated during the current study are available from the corresponding author on a reasonable request.

## Abbreviations

SCAs: Sex chromosome aneuploidies
LMP: Last menstrual period
AMA: Advanced maternal age
NIPT: Noninvasive prenatal testing
MSS: Maternal serological screening
ISCN: International system for human cytogenetic nomenclature guidelines
OR: Odds ratio
CI: Confidence interval
MPS: Massively parallel sequencing
NGS: Next-generation sequencing
ICSI: Intracytoplasmic sperm injection
IVF-ET: In vitro fertilization embryo transfer
